# Putative Role of MicroRNA-Regulated Pathways in Comorbid Neurological and Cardiovascular Disorders

**DOI:** 10.1155/2009/849519

**Published:** 2009-08-24

**Authors:** Sébastien S. Hébert

**Affiliations:** ^1^Centre de Recherche du CHUQ (CHUL), Axe Neurosciences, Canada; ^2^Département de Biologie Médicale, Université Laval, Laval, Canada QC G1k 7P4

## Abstract

*Background*. The conserved noncoding microRNAs (miRNAs) that function to regulate gene expression are essential for the development and function of the brain and heart. Changes in miRNA expression profiles are associated with an increased risk for developing neurodegenerative disorders as well as heart failure. Here, the hypothesis of how miRNA-regulated pathways could contribute to comorbid neurological and cardiovascular disorders will be discussed. *Presentation*. Changes in miRNA expression occurring in the brain and heart could have an impact on coexisting neurological and cardiovascular characteristics by (1) modulating organ function, (2) accentuating cellular stress, and (3) impinging on neuronal and/or heart cell survival. *Testing*. Evaluation of miRNA expression profiles in the brain and heart tissues from individuals with comorbid neurodegenerative and cardiovascular disorders will be of great importance and relevance. *Implications*. Careful experimental design will shed light to the deeper understanding of the molecular mechanisms tying up those different but yet somehow connected diseases.

## 1. Background

In recent years, increasing evidence suggests that genetic and epigenetic factors could be involved in disease comorbidity [1–3]. On this line of thought, Lee et al. have identified a common network of genes (and metabolic pathways) that was altered in Alzheimer's disease (AD) coinciding with myocardial infarction (MI) [1]. Indeed, increased activity of ACE and APOE genes, both involved in lipid metabolism, was significantly associated to the coexistence of these disorders. Given the central role of the genome and proteome in biology and pathobiology, it is safe to predict that misregulation of specific gene expression networks could influence disease state and in some cases comorbidity. 

 Following the discovery of microRNAs (miRNAs) in *C*.  *elegans* more than a decade ago [4], research has majorly evolved in order to gain insight of how the miRNA gene network can have an impact on health and disease in humans. Studies in animal models demonstrate that miRNA genes are essential for life [5, 6], whereas changes in miRNA expression profiles in humans are found in several diseases, such as cancer [7] as well as neurological and cardiovascular disorders (see below). 

 The miRNA gene family comprises a class of highly conserved small (~19–23 nt) nonprotein-coding RNAs that function in the cell to regulate gene expression at the posttranscriptional level [6]. Like protein-coding genes, miRNAs are embedded in the genome and generated by a multistep process ([8] and references therein). The fully processed mature miRNA molecule functions as part of the RNA-induced silencing complex (RISC) and affects gene expression. This is achieved by binding to the 3′UTR of target messenger RNAs (mRNAs), leading to their translational repression or degradation. Individual or families of miRNAs can target up to several hundred mRNAs, thus controlling complex gene expression pathways and biological systems [9]. 

 The role of miRNAs, in accordance with most biological systems, has been best studied in development. Initial studies inactivating Dicer, a type III RNase involved in miRNA maturation (and therefore function), demonstrate that miRNAs are essential for life [10]. Tissue-specific deletion of Dicer (and subsequently mature miRNAs) causes abnormal brain and heart development in both Zebrafish and mouse [11–14]. Cell-specific functional roles for miRNAs in these organs are also emerging. In detail, the brain-specific miRNA miR-134 is involved in dendritic spine formation as well as in synaptic plasticity [15], while miR-124 [16] and miR-132 [17] are implicated in neurite outgrowth. In addition, muscle-enriched miR-1 and miR-133 seem to control heart muscle growth and differentiation [18, 19] and recently shown to regulate muscle gene expression and influence sarcomere assembly [20].

## 2. Presentation of the Hypothesis

Taking into account the growing evidence that miRNAs are key regulators of gene expression and necessary for the function and maintenance of all major biological systems, it is reasonable to speculate that loss of the fine-tuning of this network could be relevant for the development of comorbid diseases. One would therefore anticipate that miRNA dysfunction occurs concomitantly (or in some cases consecutively) in the brain and heart. Obviously, nonautonomous effects should not be ruled out, since they could indirectly lead to changes in miRNA expression and function. 

 Out of the known 706 human (547 mouse) miRNAs (http://microrna.sanger.ac.uk), an abundant and particularly diverse group is expressed in the mammalian brain and heart [21–25]. Several of these miRNAs are coexpressed in both tissues (e.g., miR-29, let-7, miR-21, miR-103, miR-106, miR-17-5p) at various relative levels of expression [22, 26–28]. Interestingly, a study by Kuhn et al. showed that a subset of chromosome 21-derived miRNAs are overexpressed in both the brain and heart tissues of down syndrome patients, thus providing genetic proof-of-concept for the possibility that specific miRNAs can be coregulated in these organs [29]. It is noteworthy that despite the fact that certain miRNAs are likely more involved in tissue development (e.g., miR-106, miR-17-5p) [30], the expressions of some are specifically altered (either up- or downregulated) in disease or disease-simulated conditions in mouse models and/or in humans. Recent studies have indeed documented changes in the levels of specific miRNAs in several major neurodegenerative disorders including AD [27, 31], Huntington's disease [32], and Parkinson's disease [33]. Alterations in miRNA levels have been shown as well in various heart pathologies including arrhythmia, cardiac fibrosis, angiogenesis, and cardiac hypertrophy (reviewed in [26, 34, 35]). Whether all the reported changes in miRNA expression are involved in respective diseases is doubtful (see below) but remains to be explored. 

 Given recent findings, the hypothesis that specific variations in miRNA expression could play a significant role in comorbid neurological and cardiovascular pathologies is attractive. Alterations of a single miRNA could have profound effects on hundreds of target genes [36, 37], thus possibly implicating multiple biological pathways. However, not all reported changes in miRNA expression would necessarily be relevant to disease progression. Indeed, only a few miRNAs could exert their regulatory function directly or indirectly on disease-related genes, such as ACE and APOE. 

 Downregulation of miR-29 in AD brain as well as in MI (commonly known as heart attack) is an appealing example of how a specific miRNA (or miRNA family) network could be involved in simultaneous existence of neurological and cardiovascular pathological features. In vitro and in vivo studies performed in the mouse heart have shown that miR-29 (expressed mainly in cardiac fibroblasts) could regulate various collagens and other ECM proteins involved in cardiac remodeling after MI [38]. In the brain, it is suggested that miR-29 (expressed in both neurons and glia) could contribute to the production of toxic amyloid-*β* peptides by regulating BACE1/*β*-secretase [31]. Other studies have shown that accumulation of amyloid-*β* peptides causes degeneration of cells in the walls of blood vessels, affects vasoactivity, and improves proteolytic mechanisms, such as fibrinolysis, anticoagulation, and degradation of the ECM [39]. Of notice, changes in the ECM are linked to neurodegeneration [40] and may play a role in AD pathology [41], whereas it is suggested that collagen levels could modulate amyloid-*β* peptide toxicity in neurons [42]. Thus, the combined action of miR-29 downregulation in the brain and heart could contribute to a detrimental feedback loop between toxic amyloid-*β* production, changes in the ECM, increased cellular stress, and ultimately cell dysfunction and death. In this way, the collective effects of miR-29 downregulation in the brain and heart could significantly increase the risk for MI in AD patients and perhaps be involved in other comorbid neurological and cardiovascular disorders (such as AD and congestive heart failure). It should be noticed that the physiological role(s) of miR-29, as with most miRNAs, remains unknown. Nonetheless, it is safe to predict that changes in (single or numerous) miRNA-regulated pathways may have multiple effects on numerous cellular and biological pathways involved in neurological and cardiovascular physiology and pathophysiology, including apoptosis, lipid metabolism, and oxidative stress.

## 3. Testing the Hypothesis

Simple studies can be performed to evaluate whether a link between miRNA misexpression and neurological and cardiovascular comorbidity exists. The first step would be to analyze global miRNA expression profiles from total RNA isolated from the brain and heart tissues from (the same) individuals with coinciding characteristics of neurodegenerative and cardiovascular disorders. Several miRNA gene platforms (arrays) are commercially available. In this way, it would be possible to map the individual (i.e., brain- or heart-specific) and shared (i.e., both organs) changes in miRNA expression. Thus, heart and brain tissue banks for research on cooccurring cardiovascular and neurological/psychiatric disorders could be easily used for miRNA studies. Subsequent steps would be focused on the identification of miRNA target genes and pathways, ideally previously associated with (respective or shared) disease(s). Several bioinformatics logarithms for miRNA prediction sites in 3′UTR of genes are freely available online (*Pictar*, *miRANDA*, *TargetScan*, etc.). Functional validation of miRNA target genes in the physiological and pathological contexts may be guided through current literature (e.g., see [31]). 

 “What causes changes in miRNA expression?” Unfortunately, the underlying mechanisms of the ways and reasons miRNAs become deregulated in disease (and specifically in comorbid disease) remain largely unknown. It is possible that accumulating genetic, cellular, or environmental insults, for instance, during ageing, could affect miRNA gene expression. Some of these effects are perhaps governed themselves by transcription and/or cellular factors, since miRNAs have regulatory promoter regions and are transcribed mainly by RNA polymerase II [43, 44]. An elegant study by Wang et al. showed that, in myoblasts, the miR-29 promoter (and more particularly the miR-29b-2/miR-29c cluster) is regulated by the transcription factors NF*κ*B and YY1 [45]. During myogenesis, NF*κ*B and YY1 downregulation causes derepression of miR-29 that, in turn, accelerates differentiation by targeting its repressor YY1. Whether this complex regulatory feedback loop between miR-29, NF*κ*B, and YY1 exists in neurons remains to be explored. Interestingly, both NF*κ*B and YY1 have been involved in BACE1 regulation [46, 47], and an increase in YY1 expression was observed in human heart failure [48]. It remains therefore an interesting hypothesis that changes in NF*κ*B and/or YY1 may contribute, at least in part, to abnormal miR-29 expression in both the heart and brain. It has been shown already that decreased miR-29 correlates with increased BACE1 in AD brain [31]. Whether a similar correlation exists between NF*κ*B/YY1 and BACE1 (and perhaps ECM proteins) in these cases needs to be tested. On this line of thought, it would be interesting to see if these elements are affected in the heart of these individuals, or others, suffering from various heart conditions. It is worth mentioning that NF*κ*B seems also to be involved in the regulation of miR-146a expression and could play a role in the inflammation response in AD brain [49]. 

 Secondary (downstream) effects should also not be ignored, as comorbidity may result from a primary disease. This latter hypothesis could be tested in disease-prone animal models, where, for example, excessive soluble amyloid-*β* peptides produced in the brain of transgenic AD mouse models could induce or modulate miRNA expression in the heart.

## 4. Implications of the Hypothesis

These results could lead to the identification of common miRNAs and target genes/pathways involved in cooccurring neurological and cardiovascular disorders. The use of RNA interference and miRNA oligonucleotides into clinic could help alleviate the risk for heart failure in patients suffering from AD and possibly other neurodegenerative disorders. Concretely, miR-29 mimics can be used to reduce cardiac fibrosis, thus providing an alternative method of maintaining physiological levels of critical target genes of miR-29. Cardiac fibrosis results in stiffening of the heart, diminished contractility, and abnormalities in cardiac conductance. A beneficial effect of miR-29 mimics on BACE1 and amyloid-*β* levels in the brain (and vascular system) is equally envisaged. Reversing one or both of these processes could therefore represent an important therapeutic target of MI management and neurodegeneration. However, a word of caution is needed especially with regard to unwanted off-target effects and the multiplicity of miRNA targets. Thus, many optimization efforts will be required before miRNAs can be used into clinic.

## 5. Competing Interests

None is declared.

## List of Abbreviations

BACE1: Beta-site APP cleaving enzyme
ACE: Angiotensin I converting enzyme
APOE: Apolipoprotein E
ECM: Extracellular matrix
3′UTR: 3′untranslated region.
